# Effect of Exercise Intensity on Cell-Mediated Immunity

**DOI:** 10.3390/sports9010008

**Published:** 2021-01-11

**Authors:** Katsuhiko Suzuki, Harumi Hayashida

**Affiliations:** 1 Faculty of Sport Sciences, Waseda University, 2-579-15 Mikajima, Tokorozawa 359-1192, Japan; 2 Faculty of Culture and Sport Policy, Toin University of Yokohama, 1614 Kurogane-cho, Aoba-ku, Yokohama 225-8503, Japan; h-hayashida@fuji.waseda.jp

**Keywords:** exercise, walking, yoga, cellular immune system, cytokines, inflammation

## Abstract

Moderate-intensity exercise is considered to enhance immune function and to be useful for preventing acute upper respiratory infections and similar conditions. Many people practice low-intensity short-duration exercise with the expectation of a beneficial effect on immunocompetency. However, it is difficult to affirm the existence of definite evidence of such a benefit. In this article, we discuss the effects of low-intensity short-duration exercise on cell-mediated immunity, and contrast them to the effects of high-intensity and long-duration exercise. Whereas high-intensity exercise induces inflammation and reduces cell-mediated immune system function, low-intensity exercise does not appear to have a large effect on either inflammation or cell-mediated immune function. Low-intensity exercises such as walking and yoga, which are helpful to relieve stress, cannot be considered as harmful to the immune system. Although yoga was shown to impose fewer restrictions on breathing and physical strain, the evidence that yoga enhances cell-mediated immunity remains insufficient. Therefore, further studies are needed to examine the exercise mode that may be most effective for improvement of immune functions.

## 1. Introduction

It is well known that exercise promotes cell-mediated immune system function by releasing anti-inflammatory cytokines (IL-4, IL-1ra, IL-5) at the site of injuries [1]. Further, exercise intensity and duration have been demonstrated to have a great impact on immune system function [2,3,4,5,6]. This beneficial effect of exercise is due to the anti-inflammatory effects of exercise mediated by the downregulation of Toll-like receptors and/or cytokines [6].

Exercise intensity is defined as the amount of energy expended per unit of time due to the exercise performed. Moderate-intensity exercise is considered to enhance immune function and to prevent acute upper respiratory infections and similar conditions [7,8]. Excessive amounts of prolonged high-intensity exercise may cause a downregulation of immune function. Low-intensity exercise is good for the beginner or individuals who have chronic health issues. According to a health survey conducted in the USA, 51% of respondents practiced yoga as a low-intensity short-duration exercise, and although 50% of respondents claimed a beneficial effect on the immune system as a reason to practice yoga [9], it is difficult to affirm the existence of definite evidence at this point from recent reviews [9,10]. Some other examples of low-intensity exercise are light walking, stretching or swimming at a slow and steady pace. Low-intensity short-duration exercise may have a beneficial effect on cell-mediated immune system function [3,8]. In this review paper, we discuss the effects of cytokine dynamics that result from low-intensity short-duration exercises on cell-mediated immunity, in contrast to the effects of high-intensity and long-duration exercise.

## 2. Function and Control Mechanism of Cell-Mediated Immunity

Immunity is a host defense function that aims to prevent abnormal cells such as tumor cells and foreign microorganisms from invading the body, and involves actions from several types of immune cells. Cell-mediated immunity refers to the elimination mechanisms of tumor cells and intracellular pathogens, including viruses, by the functions of immune cells, mainly comprising T cells, macrophages and natural killer (NK) cells. In the broader sense, the defense mechanisms mediated by other immune cells such as neutrophils can be included in the definition of cell-mediated immunity, since multiple immune cells exert similar effects owing to the shared bioactive substances (cytokines) that control these immune cells.

**Figure 1:**
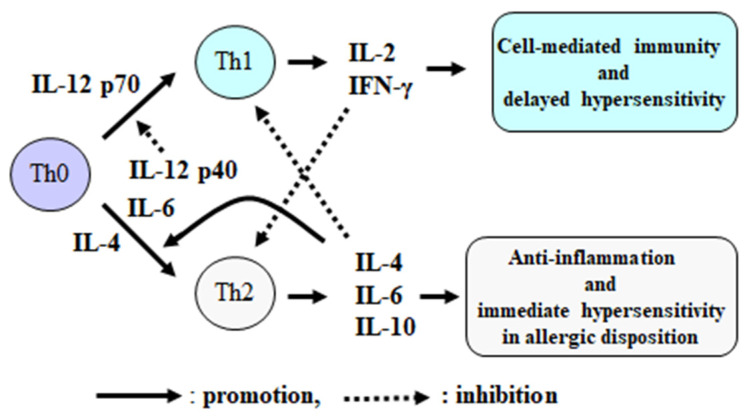
Th1/Th2 cytokine balance and immune responses. Modified from Suzuki, K., Exercise Immunol. Rev. 8: 6–48, 2002 [11].

Helper T (Th) cells, which play a central role in cell-mediated immunity, are induced to differentiate into type 1 helper T (Th1) cells by interleukin (IL)-12, which then activates cytotoxic T (Tc) cells, macrophages and NK cells via immunomodulatory cytokines such as IL-2 and interferon-gamma (IFN-γ) [11]. In contrast, type 2 helper T (Th2) cells are induced to differentiate by the anti-inflammatory cytokine IL-4 and produce IL-10 with strong immunosuppressive effects. This, in turn, suppresses cell-mediated immunity Th1 cells, which includes those which result in immune suppression or the elimination of unnecessary immune and inflammatory responses after removal of foreign bodies (Figure 1).

IL-12 is also known as a NK cell-activating factor, which has two subunits, p35 and p40; p35 exists as a p70 dimer and activates both NK cells and Th1 cells [11,12]. The IL-12 p40 subunit has 200-fold lower activity compared to that of p70 and is considered to have an inhibitory effect to IL-12 p70 [13]. IL-12 p40 has a similar structure to that of pro-inflammatory cytokines such as IL-6 to induce inflammation [12,13,14]. Therefore, cell-mediated immunity is regulated by a complex mechanism in connection to inflammation (Figure 1).

## 3. Effects of High-Intensity and Long Duration Exercise on Immune Variables

Reports demonstrate the effects of relatively high-intensity exercise and long-duration activities, such as marathon running, on immune function [15,16]. They state that immune function, which includes cell-mediated immunity, is suppressed for a few hours to a few days after such high-intensity exercise and leads to increased susceptibility to infections [15,16,17,18,19]. Cell-mediated immunity is evaluated in vivo by a delayed-onset hypersensitivity reaction after vaccination, in which one study demonstrated decreased hypersensitivity after exhaustive endurance exercise [17]. Although moderate-intensity exercise is generally considered to activate immune functions given a temporary elevation in NK cell activity, which then returns to the pre-exercise state after the exercise, NK cell activity is suppressed for several hours after high-intensity exercise [20]. Since this period of reduced immunity can provide an opportunity for pathogen invasion, the “open window model” was proposed to describe the phenomena of suppression of immune functions that depend on the intensity of endurance exercise [16,18,20,21].

The mechanism for the open window model has been speculated to involve the secretion of stress hormones, including cortisol, due to high-intensity exercise, along with IL-10, and IL-4 and IL-12 p40 that all suppress immune function [12,16,18,20,21,22]. Indeed, long-duration high-intensity exercise was shown to suppress the production of immunomodulatory cytokines [21,22,23]. In contrast, endurance exercise causes an increase and activation of blood neutrophils; however, this might be considered as an excessive biological reaction that leads to muscle and organ damage and systemic inflammation rather than to the activation of immunocompetence [23]. Such adverse reactions have not been observed after low-intensity short-duration exercise. It is believed that such exercise might not cause inflammation or immunosuppression. Therefore, such exercise is recommended, especially for those with low physical fitness or underlying chronic health conditions.

## 4. Effects of Moderate-Intensity Exercise and Training on Immune Variables

Regular moderate-intensity exercise reduces the risk of infection compared to a sedentary lifestyle. The J-curve model has been proposed in the field of exercise immunology, which indicates the risk of upper respiratory tract infection (URTI) is lower in individuals with a habit of regular moderate-intensity exercise compared to those with a sedentary lifestyle, whereas long-duration high-intensity exercise could increase the risk of infections [16,17,18,19,20,21,22,23,24]. There has been keen interest in physical activity which induces an anti-inflammatory response due to its preventive/improvement effects on chronic inflammatory diseases, including metabolic syndrome [6,22,25,26]. However, long-duration high-intensity training results in not only fatigue but also inflammation, leading to an “open window” state for pathogen invasion as described above. Since IL-10 is produced by both the innate and adaptive immune systems including monocytes, macrophages, dendritic cells, B cells, CD8+ T cells, regulatory T cells, Th1 cells and Th17 cells, it has been termed as the cytokine synthesis inhibitory factor and acts as a down regulator in response to inflammation or injury [27]. A previous study showed athletes with elevated levels of Th2 cytokines, including IL-10, during the high-intensity training period were more susceptible to infections compared with those whose Th2 cytokines did not increase [28], which suggests that IL-10 levels might increase depending on the training load and conditions [16,22,28]. Another study reported that athletes with URTI symptoms had higher IL-10 production than subjects free from URTI symptoms [29].

We further investigated exercise-induced changes in blood inflammatory markers in sedentary female college students [30,31]. In one study, participants performed 60 min of exercise on a bicycle ergometer at 75% of ventilatory threshold (VT: determined by gas exchange parameters) during different phases of the menstrual cycle (menstrual, follicular and mid-luteal phases) [30]. Blood samples were taken for IL-6 and calprotectin (monocyte/macrophage activation marker) at pre-, immediately post- and 30 min post-exercise. Both IL-6 and calprotectin concentrations were significantly increased by low-intensity exercise. There were no differences in IL-6 and calprotectin due to exercise in different phases of the menstrual cycle [30]. However, a positive correlation was found between exercise-induced changes in plasma IL-6 and calprotectin levels only in the menstrual phase [30]. This suggests that exercise at an intensity greater than 75% VT may promote an inflammatory response in the menstrual phase of the menstrual cycle.

**Figure 2:**
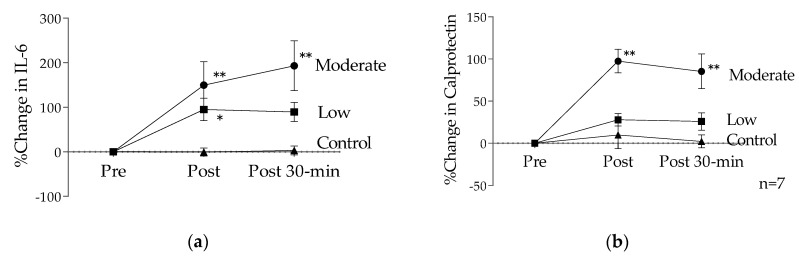
The effects of exercise intensity during the menstrual period: (**a**) % change in IL-6; (**b**) % change in calprotectin. Data are presented as means ± SE. * *p* < 0.05 versus the control trial, ** *p* < 0.001 versus the control trial.

In another study, participants performed 60 min of exercise on a cycle ergometer at VT (moderate intensity) during the menstrual phase [31], and IL-6 and calprotectin concentrations were measured. Plasma IL-6 and calprotectin concentrations were elevated post-exercise (unpublished data). During the menstrual period, low-intensity exercise induced an increase above resting values in IL-6 immediately after exercise (95%) and 30-min post-exercise (89%) [30] and moderate-intensity exercise induced a greater increase immediately after exercise (190%) and 30-min post-exercise (189%) [31]. Plasma calprotectin concentrations were significantly higher than that of pre-exercise immediately after exercise (low: 28%, moderate: 108%) and 30-min post-exercise (low: 26%, moderate: 87%), indicating intensity-dependent changes (Figure 2). Although VT intensity exercise is not an exhaustive exercise, it was found that circulating IL-6 and calprotectin continues to increase after exercise during the menstrual period. Therefore, high-intensity exercise is not recommended, especially during the menstrual period of sedentary females in terms of systemic inflammation.

## 5. Effects of Short-Duration Low-Intensity Exercise and Training on Immune Variables

As previously stated, low-intensity exercise might be most suitable for those with chronic health conditions and newcomers to exercise training. In an earlier study we investigated the effects of acute and chronic low-intensity short-duration exercise on immune parameters in healthy college students without the habit of regular exercise [8]. The participants walked on a treadmill (6 km/h) for 30 min daily, six times per week for three weeks. Lymphocyte subset, T-cell mitogenic activity, NK cell activity and blood cytokine concentrations were investigated. There were no significant differences before and after acute exercise, suggesting that low-intensity exercise might not cause changes in the immune system. However, after the three-week training period, IL-12 p40 level, which is considered to be a substance that suppresses cell-mediated immunity, significantly decreased, suggesting the possibility of induction of a Th1-dominant cytokine balance, with no significant effects on the other parameters measured listed above [8].

Besides, other researchers have documented that regular low-intensity exercise can reduce lymphocyte apoptosis and increase T-cell function, resulting in improvement of immune function [32]. Furthermore, 24 weeks of exercise improves the immune system, suggesting that the body adapts to physiological conditions [33]. Furthermore, moderate-intensity exercise can increase T-cell function, reduce inflammation and increase IL-10 [34,35,36,37]. On the other hand, strenuous exercise can increase inflammation or stress in the body, depress the immune system and increase the risk of infection [6,16,21,23,38]. Pro- and anti-inflammatory cytokines are released with intensity during exercise [11,22,39,40]. It was also reported that even moderate-intensity exercise for more than 60 min can increase inflammation in the body, whereas moderate-intensity exercise for less than 60 min with adequate rest can prevent inflammation [5,16,40]. Indeed, moderate exercise interventions stimulate anti-inflammatory and antioxidant responses, and may prevent development of many chronic diseases and comorbidities [25,41].

Yoga is a low-intensity exercise which is known to improve strength and flexibility. A recent meta-analysis on the effects of mind–body therapy on the immune system indicated that suppressive effects on blood inflammatory markers (e.g., C-reactive protein, IL-6) could be expected, whereas no remarkable effects were observed for other immune indices [42]. Practicing yoga alongside other moderate- and high-intensity exercise also modulates pro-inflammatory cytokines such as IL-6 and TNF-α [43]. A review article on yoga showed that it is a useful practice to improve mental and physical conditions, but did not address the effects on cell-mediated immunocompetence other than cytokines, and highlighted the need for a randomized controlled trial (RCT) on the topic [9]. Interestingly, a recent RCT that investigated the effects of yoga for three months suggested involvement of brain-derived neurotrophic factor (BDNF) with respect to changes in cytokines, demonstrating an increase in blood IL-1β, IL-6, IL-8, IL-10, IFN-γ and TNF-α concentrations, and a decrease of IL-12 concentration [44]. Another study which investigated the effects of yoga for three months in healthy college students further demonstrated that increases in blood IL-2 and IFN-γ concentrations were associated with improvements in oxidative stress, which suggests the possibility of activation of cell-mediated immunity [45]. In a study on the effects of 12-week yoga practices on stress during academic examinations done by medical students, the authors found that exercise led to attenuation of the increase in psychological stress parameters and a decrease in serum IFN-γ and IFN-γ/IL-4 ratios [46]. These findings suggest the possibility of activation of cell-mediated immunity. Another study that investigated the effect of yoga for 4 months on industrial workers reported a decrease of IL-1β levels and an increase of IL-10 levels, which suggests yoga had an anti-inflammatory effect on those who were exposed to pollutants and under inflammatory conditions along with the general anti-inflammatory actions of performing physical activity described above [47]. However, there is still no established consensus on these benefits. In these previous studies, the sample sizes, experimental conditions and measurement variables were inconsistent, and the authors indicated the need for further examination in more subjects in the future along with establishment of an appropriate control group. A recent meta-analysis on the effects of yoga on the immune function indicated that yoga reduced levels of inflammatory cytokines (IL-1β) in healthy individuals and clinical populations [10]. In addition, no studies reported increases in pro-inflammatory markers or decreases in anti-inflammatory markers, suggesting that yoga might not adversely affect immune function [10].

## 6. Conclusions and Challenges for the Future

Low-intensity exercise such as walking does not appear to largely affect cell-mediated immunocompetency in both regular and sporadic physical activity [7,8]. Even moderate-intensity exercise in hot environments [48,49,50], during menstruation, etc., can cause inflammation. While low-intensity exercise such as yoga, which may help relieve stress, cannot be considered harmful to the immune system, they should be performed in order to avoid too large an exercise load for those with poor physical fitness or with underlying health conditions. Although yoga imposes fewer restrictions on breathing and physical strain with efficacy for the humoral defense system [51,52], the evidence that yoga enhances cell-mediated immunocompetency remains insufficient. Therefore, further studies are needed to identify differential measurements of IL-12 p40/p70 along with oxidative stress markers in more appropriate experimental designs to best examine the exercise intensity that may be most effective for improvement of immune function [6,11,50,53].
